# Insecticidal activity of Simparica and Simparica Trio against *Aedes aegypti* in dogs

**DOI:** 10.1186/s13071-023-05699-z

**Published:** 2023-03-09

**Authors:** Thomas Geurden, Sara Chapin, John W. McCall, Abdelmoneim Mansour, Sean P. Mahabir, Kristina Kryda, Tom McTier

**Affiliations:** 1 Zoetis Incorporated, Mercuriusstraat 20, 1390 Zaventem, Belgium; 2 Zoetis Incorporated, Kalamazoo, MI 49007 USA; 3 TRS Labs, Incorporated, Athens, GA 30607 USA

**Keywords:** *Aedes aegypti*, Dogs, Insecticidal, Isoxazoline, Mosquito, Efficacy, Sarolaner

## Abstract

**Background:**

*Aedes aegypti* is one of the main species responsible for the transmission of mosquito-borne pathogens worldwide. The isoxazoline Sarolaner has excellent efficacy as an acaricide against ticks and mites and as an insecticide against fleas, and potential efficacy against other insects.

**Methods:**

In each of two laboratory studies, 24 dogs were randomly allocated (*n* = 8/group) to an untreated control group, a Simparica-treated group (at the minimum dose of 2.0 mg/kg sarolaner), or a Simparica Trio-treated group (at the minimum dose of 1.2 mg/kg sarolaner, 24 µg/kg moxidectin and 5 mg/kg pyrantel), based on pre-treatment mosquito counts. Treatments were administered orally once on day 0. Each dog was exposed to 50 unfed female adult *A. aegypti* mosquitoes for 1 h on days 1, 7, 14, 21, 28 and 35. After each exposure, mosquitoes were counted for each dog and characterized as live, moribund or dead, and as fed or unfed. Dead mosquitoes were counted and removed at 12, 24 and 48 h post-exposure in study 1 and at 24, 48, 72, 96 and 120 h post-exposure in study 2. In study 2, mosquito eggs were collected from 72 h post-exposure until 120 h post-exposure. Insecticidal efficacy was calculated based on the reduction of the arithmetic mean live fed-mosquito counts in each of the treated groups versus the untreated control group for every timepoint post-exposure.

**Results:**

Adequate challenge was demonstrated in both studies, with arithmetic mean live fed-mosquito counts ranging from 35.5 to 45.0 for the untreated group. Mean mosquito counts for dogs treated with Simparica and Simparica Trio were significantly (*P* < 0.0001) reduced within 48 h after exposure on all study days. In study 1, Simparica treatment provided ≥ 96.8% reduction in the arithmetic mean live fed-mosquito counts for 28 days, and Simparica Trio treatment provided ≥ 90.3% reduction for 21 days. In study 2, Simparica treatment provided ≥ 99.4% reduction for 35 days (from 48 h onwards), and Simparica Trio treatment provided ≥ 97.8% reduction for 28 days (from 72 h onwards).

**Conclusions:**

Both studies demonstrated that a single oral dose of Simparica or Simparica Trio provides high efficacy against mosquitoes in dogs within 24–72 h after exposure for an entire month.

**Graphical Abstract:**

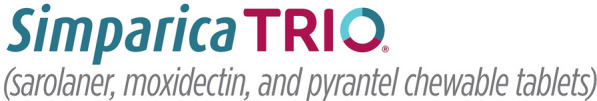

## Background

*Aedes aegypti* is reported globally and is one of the main species responsible for the worldwide transmission of mosquito-borne pathogens, including *Dirofilaria immitis* and *Dirofilaria repens* in dogs [1]. In addition to its role in the transmission of vector-borne diseases, the bites of *A. aegypti* cause discomfort and irritation, and can lead to hypersensitivity reactions in some animals [2].

The isoxazoline Sarolaner has excellent efficacy against fleas, ticks, and mites through its antiparasitic activity via specific blockade of insect and acarid γ-aminobutyric acid- and glutamate-gated chloride channels. The safety of sarolaner has been evaluated in mouse models as well as through specific target animal safety studies [3]. Furthermore, isoxazolines have been reported to have insecticidal efficacy against mosquitoes [4–6]. Systemically administered isoxazolines have the potential to reduce the mosquito population in the microenvironment (e.g. household) around the treated animal by killing the mosquitoes prior to a new feeding bout. Hence, systemically administered isoxazolines have an indirect effect on the rate of vector-borne pathogen transmission within that microenvironment, although the mosquitoes must blood-feed for exposure, and thus insecticidal efficacy might not directly prevent pathogen transmission to the treated animal. While prevention of mosquito bites is usually achieved through the use of repellent molecules, the use of a systemic product as a tool in vector control has previously been described for the control of malaria [7], as it reduces the survival of vectors that feed on host populations that have been previously treated. The removal of adult vectors from the area also precludes their reproduction, which may otherwise lead to an exponential increase in the vector’s population. Sarolaner has been shown to be effective against *A. aegypti* in in vitro assays [6], and its insecticidal activity (Simparica and Simparica Trio) against *A. aegypti* was evaluated in an animal model in the laboratory studies reported here.

## Methods

### General study design and animal management

Two negative-controlled laboratory studies were conducted. In each study, 26 male and female purpose-bred beagles were acclimatized to the study site 7–21 days prior to treatment. The dogs ranged from 6 to 9 months of age and from 5.2 to 9.2 kg in weight. The dogs were identified by uniquely numbered ear tattoos and housed individually in indoor enclosures in a mosquito-proof facility that conformed to accepted animal welfare guidelines and ensured that there was no direct contact between them. Pre-treatment mosquito infestations and mosquito counts were performed 5 days prior to dosing (day -5) for all 26 dogs, and the two with the lowest number of fed mosquitoes were excluded from the study. The remaining 24 dogs were allocated to one of three treatment groups in each block (total of eight dogs per treatment group) according to a randomized complete block design based on pre-treatment mosquito counts: untreated group (T01), Simparica-treated group (sarolaner; (T02), and Simparica Trio-treated group [sarolaner, moxidectin, pyrantel (as pamoate salt); T03]. For treatment groups T02 and T03, tablets of varying strengths were used such that a combination of tablets could be administered to ensure that the dogs were appropriately dosed at the low end of the label dosage without underdosing. For T02, the minimum target dose was 2.0 mg/kg sarolaner (actual doses ranged from 2.0 to 2.9 mg/kg). For T03, the minimum target dose was 1.2 mg/kg sarolaner (actual doses ranged from 1.2 to 1.6 mg/kg), 24 µg/kg moxidectin (actual doses ranged from 24 to 32 µg/kg) and 5.0 mg/kg pyrantel (actual doses ranged from 5.0 to 6.6 mg/kg). The tablet(s) were administered orally, and food was withheld overnight (at least 12 h) prior to treatment administration; the dogs were not fed again until at least 4 h post-treatment.

Procedures for animal use, as described in the study protocol, were approved by the site’s Institutional Animal Care and Use Committee prior to the start of the study. Only healthy (as determined by the study veterinarian), not pregnant or lactating animals, which were not intended for breeding were considered for inclusion in the study. Dogs were not allowed to be treated with ProHeart 6 and/or ProHeart 12 at any time prior to the study or with a macrocyclic lactone within 90 days before the study started, and all the dogs underwent a sufficiently long wash-out period to ensure that no residual ectoparasiticide efficacy remained from any previously administered compounds. Once enrolled in the study, any dog that, in the view of the investigator or an appropriately experienced veterinarian, showed excessive discomfort due to disease or an adverse reaction, or suffered from a life-threatening illness, was to be removed from the study and to receive appropriate veterinary treatment. All of the study dogs were housed in 1.85-m^2^ runs with a sloped concrete floor, and aluminum front and side panels. Housing was in compliance with the Animal Welfare Act 9 CFR parts 1–4. All dogs were moved to the study facilities on or before day -14. Dogs were moved into their allocated pens on or before day -1. On day 0, prior to treatment, as well as at 1 h, 3 h, 6 h and 24 h after dosing, all dogs were assessed for general health. Throughout the study (day -14 to day 35), the general health of the dogs was assessed by appropriately trained personnel at least twice daily. These observations were recorded and are summarized in the study report.

### Mosquito infestations and counts

The *A. aegypti* isolate was obtained from the Department of Parasitology and Entomology at the Liverpool School of Tropical Medicine, UK, in 1972. TRS Labs, Incorporated obtained this isolate from the University of Georgia in 1980. During the years that the mosquitoes were maintained at University of Georgia and then at TRS Labs, Incorporated, both laboratory colonies were refreshed with eggs from the other colony.

Post-treatment mosquito infestations were performed on days 1, 7, 14, 21, 28 and 35. For each infestation, dogs were sedated with Dexdomitor at 0.04 mL/kg and Butorphanol at 0.02 mL/kg, or Dexdomitor at 0.04 mL/kg and Butorphanol at 0.02 mL/kg plus Antisedan at 0.15 mL/kg to prevent mosquito-bite hypersensitivity reactions, and placed into individual infestation chamber into which 50 ± 5 unfed female adult *A. aegypti* mosquitoes were released. After 60 ± 10 min of exposure, all live mosquitoes were removed from the infestation chamber and the dogs were then carefully taken out of the chamber to allow for removal of the dead mosquitoes. All dead mosquitoes collected from the infestation chamber were then counted. All fed live and moribund mosquitoes in the infestation chamber were aspirated into separate incubation cartons (one chamber per animal) using a vacuum pump, and were counted and evaluated for feeding status. Other mosquitoes (dead, and live unfed mosquitoes) were discarded. The live fed mosquitoes were kept in an incubation carton which had a nylon screen mesh top. On the tops (lids) of the incubation cartons, the mosquitoes had cubes of sugar and cotton soaked with sugar water at their disposal. Dead mosquitoes were counted at 12 ± 2 h, 24 ± 2 h and 48 ± 2 h after exposure to the animals (study 1) or at 24 ± 2 h, 48 ± 2 h, 72 ± 2 h, 96 ± 2 h, and 120 ± 2 h after exposure (study 2). Dead mosquitoes were counted after they had been removed from the incubation carton at each time point, while the live/moribund mosquitoes remained in the carton until after the last observation had been made.

During the counts, the mosquitoes were categorized as live, moribund, or dead and as fed or unfed. A mosquito was considered live when it exhibited normal behaviour, such as being capable of walking or flying. A mosquito was considered moribund if it was unable to perform normal locomotion and exhibited clear signs of neurological disruption, such as showing a lack of balance or being unable to fly in response to external stimuli. The feeding status of live or moribund mosquitoes was determined with the naked eye according to distension of the abdomen and the presence of blood in the abdomen. Dead mosquitoes were assessed for feeding status by placing each of them on tissue paper and squashing the abdomen with a spatula or other suitable object to assess if a blood meal had been taken.

### Mosquito egg counts

In study 2, mosquito egg counts were conducted on each study day, i.e. study days 1, 7, 14, 21, 28 and 35, at 72 h, 96 h and 120 h post-exposure. Flat cotton discs (55 mm inner diameter; Greenwise Hypoallergenic Cotton Rounds) were placed in a pan of deionized water and allowed to soak overnight. At 72 ± 2 h after exposure, the time allotted to allow for mosquito egg laying, a water-soaked cotton disc was placed on the top of each incubation carton and the lid of a small petri dish (60 × 15 mm) was placed over the disc. Thirty minutes later, the cotton disc was removed, placed in an individually labelled petri dish with a lid (100 × 20 mm) and transferred to the insectary (~ 27 °C, 80% relative humidity). Immediately after removing the first disc, a second water-soaked disc was placed on each incubation carton. Mosquitoes were allowed to lay eggs for another 24 h (up to 96 h post-exposure), after which the cotton disc was removed and replaced with another cotton disc for an additional 24-h time period for egg collection (up to 120 h post-exposure). The eggs collected on the cotton discs were counted as follows: the egg collection disc was placed egg-side up and the percentage of the egg-collection disc covered by eggs was estimated. If the area of the egg-collection disc covered with eggs was estimated to be ˂30% of the total area, all the eggs on the disc were counted by microscopic examination. If the area of the egg-collection disc covered with eggs was estimated to be > 30% of the total area, a randomized, standard fraction of the disc was examined for egg counts, and the appropriate calculations were made to estimate the total number of eggs on the disc.

### Statistical analysis

The primary endpoint for these studies was the live fed-mosquito count defined as the combination of live fed-mosquito and moribund fed-mosquito counts. For each post-treatment infestation (days 1, 7, 14, 21, 28 and 35), the number of live fed mosquitoes at a given time point after exposure was calculated as the difference between the number of live fed mosquitoes at the previous timepoint and the number of dead mosquitoes at that timepoint. The percent efficacy in the treatment groups compared to the untreated control group for each post-treatment infestation (days 1, 7, 14, 21, 28 and 35) was calculated separately for the counts at the different timepoints using the following formula: [(*C* –* T*) /* C*] × 100, where* C* is the arithmetic mean live fed-mosquito count for the untreated control group and* T* is the arithmetic mean live fed-mosquito count for the treatment group. A mixed linear model with treatment group as a fixed effect and block and error as random effects at each time point for live fed-mosquito counts was used. Testing was two sided at the significance level α = 0.05. The egg counts were summarized.

## Results

### Health observations

There were no treatment-related adverse events during these studies. In both studies, dogs from all treatment groups experienced post-infestation mosquito bite reactions such as chemosis and periocular swelling, in addition to sporadic events commonly observed in laboratory dogs, which included various dermatologic and gastrointestinal abnormalities.

### Study 1

The arithmetic mean mosquito counts, the range of mosquito counts, the percent efficacy and the statistical comparisons against the untreated control group for the number of live fed mosquitoes at 12 h, 24 h and 48 h post-exposure for the different infestation days (days 1, 7, 14, 21, 28 and 35) are shown in Table 1.

**Table 1:** Range, arithmetic mean live fed-mosquito counts and percent efficacy relative to the untreated control group at different timepoints (12 h, 24 h and 48 h) after exposure for all study days for dogs treated orally with Simparica or Simparica Trio in study 1

			Study day					
Time post-exposure (hours)			1	7	14	21	28	35
12	Untreated control	Range	34–50	39–46	32–52	24–53	15–56	34–52
		Arithmetic mean	43.0	42.0	41.4	38.5	44.3	42.3
	Simparica	Range	0–10	0–0	1–18	15–44	35–48	30–44
		Arithmetic mean	3.0	0.0	8.5	34.0	43.0	39.8
		% Reduction	93.0*	100*	79.5*	11.7	2.8	5.9
	Simparica Trio	Range	0–14	0–17	23–43	29–55	33–49	38–50
		Arithmetic mean	6.6	2.1	34.6	41.5	44.5	44.4
		% Reduction	84.6*	94.9*	16.3	-7.8	-0.6	-5.0
24	Untreated control	Range	32–49	39–46	32–52	24–53	15–56	34–52
		Arithmetic mean	42.8	42.0	41.4	38.5	41.8	42.3
	Simparica	Range	0–5	0–0	0–3	0–14	13–42	23–44
		Arithmetic mean	1.4	0.0	0.8	8.5	27.5	35.3
		% Reduction	96.8*	100*	98.2*	77.9*	34.1*	16.6*
	Simparica Trio	Range	0–14	0–0	0–9	11–35	33–49	38–50
		Arithmetic mean	4.1	0.0	3.8	22.6	43.1	44.0
		% Reduction	90.4*	100*	90.9*	41.2*	-3.3	-4.1
48	Untreated control	Range	32–49	39–46	32–52	24–53	15–56	34–52
		Arithmetic mean	42.4	41.8	41.3	38.3	42.7	42.1
	Simparica	Range	0–5	0–0	0–1	0–2	0–4	0–14
		Arithmetic mean	1.4	0.0	0.1	0.4	0.6	5.0
		% Reduction	96.8*	100*	99.7*	99.0*	98.5*	88.1*
	Simparica Trio	Range	0–14	0–0	0–1	0–5	0–48	4–45
		Arithmetic mean	4.1	0.0	0.4	1.1	12.8	16.9
		% Reduction	90.3*	100*	99.1*	97.1*	70.2*	59.9*

* *P* < 0.05 (significantly different from the placebo)

The untreated control group consistently maintained high live fed-mosquito counts, demonstrating adequate challenge throughout the study, with arithmetic mean counts ranging from 38.3 to 44.3. In the T02 group (Simparica), the arithmetic mean live fed-mosquito counts were significantly lower than those in the untreated control group on days 1, 7 and 14 (*P* < 0.0001) 12 h post-exposure, and on all days 24 h (*P* ≤ 0.0365) and 48 h (*P* < 0.0001) post-exposure. In the T03 group (Simparica Trio), the arithmetic mean live fed-mosquito counts were significantly lower than those in the untreated control group on days 1, 7 and 14 (*P* ≤ 0.0444) 12 h post-exposure, on days 1,7, 14, and 21 (*P* < 0.0001) 24 h post-exposure, and on all days (*P* < 0.0001) 48 h post-exposure.

### Study 2

The arithmetic mean mosquito counts, the range of mosquito counts, the percent efficacy and the statistical comparisons against the untreated control group for the number of live fed-mosquitoes at 24 h, 48 h, 72 h, 96 h and 120 h post-exposure for the different infestation days (days 1, 7, 14, 21, 28 and 35) are shown in Table 2.

**Table 2:** Range, arithmetic mean live fed-mosquito counts and percent efficacy relative to the untreated control group at the different timepoints (24 h, 48 h, 72 h, 96 h and 120 h) after exposure for all study days for dogs treated orally with Simparica or Simparica Trio in study 2

Time post-exposure (hours)			Study day					
			1	7	14	21	28	35
24	Untreated control	Range	35–46	21–47	37–49	36–48	42–50	35–50
		Arithmetic mean	40.8	37.1	42.9	43.5	46.3	42.0
	Simparica	Range	0–8	3–6	2–8	0–9	3–14	7–43
		Arithmetic mean	4.1	4.3	5.8	4.9	8.6	20.3
		% Reduction	89.9*	88.6*	86.6*	88.8*	81.4*	51.8*
	Simparica Trio	Range	3–9	0–6	3–8	4–12	12–46	8–45
		Arithmetic mean	5.4	2.9	4.8	7.5	25.5	31.9
		% Reduction	86.8*	92.3*	88.9*	82.8*	44.9*	24.1
48	Untreated control	Range	35–46	19–46	35–49	35–48	41–50	35–50
		Arithmetic mean	40.8	36.3	42.3	43.1	45.8	41.9
	Simparica	Range	0–0	0–0	0–2	0–1	0–1	0–3
		Arithmetic mean	0.0	0.0	0.4	0.3	0.3	0.5
		% Reduction	100*	100*	99.1*	99.4*	99.5*	98.8*
	Simparica Trio	Range	0–1	0–1	0–1	0–1	0–32	0–42
		Arithmetic mean	0.1	0.1	0.1	0.3	5.1	15.1
		% Reduction	99.7*	99.7*	99.7*	99.4*	88.8*	63.9*
72	Untreated control	Range	35–46	19–46	34–49	35–48	40–50	35–50
		Arithmetic mean	40.6	36.1	42.1	43.0	45.4	41.9
	Simparica	Range	0–0	0–0	0–2	0–1	0–1	0–1
		Arithmetic mean	0.0	0.0	0.4	0.3	0.3	0.1
		% Reduction	100*	100*	99.1*	99.4*	99.4*	99.7*
	Simparica Trio	Range	0–1	0–1	0–1	0–1	0–6	0–36
		Arithmetic mean	0.1	0.1	0.1	0.3	1.0	9.3
		% Reduction	99.7*	99.7*	99.7*	99.4*	97.8*	77.9*
96	Untreated control	Range	35–46	19–46	34–49	35–48	40–50	34–50
		Arithmetic mean	40.5	35.8	41.5	42.8	45.4	41.4
	Simparica	Range	0–0	0–0	0–2	0–1	0–1	0–1
		Arithmetic mean	0.0	0.0	0.4	0.3	0.3	0.1
		% Reduction	100*	100*	99.1*	99.4*	99.4*	99.7*
	Simparica Trio	Range	0–1	0–1	0–1	0–1	0–3	0–29
		Arithmetic mean	0.1	0.1	0.1	0.3	0.6	7.8
		% Reduction	99.7*	99.7*	99.7*	99.4*	98.6*	81.3*
120	Untreated control	Range	35–46	19–46	34–49	34–48	37–50	34–49
		Arithmetic mean	40.5	35.8	41.5	42.3	44.8	41.1
	Simparica	Range	0–0	0–0	0–2	0–1	0–1	0–1
		Arithmetic mean	0.0	0.0	0.4	0.3	0.3	0.1
		% Reduction	100*	100*	99.1*	99.2*	99.4*	99.7*
	Simparica Trio	Range	0–1	0–1	0–1	0–1	0–3	0–28
		Arithmetic mean	0.1	0.1	0.1	0.3	0.7	7.6
		% Reduction	99.7*	99.7*	99.7*	99.4*	98.4*	81.5*

* *P* < 0.05 (significantly different from the placebo)

The untreated control group consistently maintained high live fed-mosquito counts, demonstrating adequate challenge throughout the study, with arithmetic mean counts ranging between 35.8–46.3. In the T02 group (Simparica), the arithmetic mean live fed-mosquito counts were significantly (*P* < 0.0001) lower than those in the untreated control group on all days at all timepoints post-exposure. In the T03 group (Simparica Trio), the arithmetic mean live fed-mosquito counts were significantly lower than those in the untreated control group on all days (*P* < 0.0001) at all timepoints post-exposure, except for 24 h on day 35.

### Egg counts

The results of the egg counts are provided in Table 3.

**Table 3:** The mosquito egg counts (mean, minimum and maximum) at the different count times (72 h, 96 h and 120 h) after exposure on the different study days in the different treatment groups; the number of animals with a positive egg count is shown

Study day	Treatment	Time post-exposure (hours)	No. of animals	Mean	Minimum	Maximum
1	Untreated control	72	8	392.6	16	926
		96	8	425.6	217	725
		120	8	76.9	0	277
7	Untreated control	72	8	485.1	276	799
		96	8	362.0	131	783
		120	8	45.0	0	96
14	Untreated control	72	8	401.9	328	565
		96	8	806.6	167	1546
		120	8	91.9	0	329
21	Untreated control	72	8	468.4	314	735
		96	8	718.0	283	1152
		120	8	118.4	21	178
28	Untreated control	72	8	497.0	406	584
		96	8	1166.4	333	2417
		120	8	59.8	8	196
	Simparica Trio	72	1	70.0	70	70
		96	1	153.0	153	153
		120	1	2.0	2	2
35	Untreated control	72	8	1153.1	776	1755
		96	7	369.6	292	429
		120	8	43.5	0	284
	Simparica Trio	72	3	428.7	331	591
		96	4	385.0	23	962
		120	4	74.0	0	153

In the untreated control group, the mosquitoes collected from all animals laid eggs on all study days, whereas in the Simparica-treated group (T02), no eggs were collected on any study day. In the Simparica Trio-treated group (T03), eggs were collected from mosquitoes from one dog on day 28 and four dogs on day 35. The highest egg counts were observed at 72 h (disc removed after 30 min) and 96 h (second disc removed after 24 h); the mean egg count declined towards 120 h.

## Discussion

There were no treatment-related adverse events during these studies, which is in line with the safety profile of sarolaner [3]. The results of both studies confirm the high insecticidal efficacy of sarolaner against *A. aegypti* at the minimum dose of 2.0 mg/kg for Simparica or at the minimum dose of 1.2 mg/kg sarolaner, 24 µg/kg moxidectin and 5 mg/kg pyrantel for Simparica Trio. In study 1, efficacy was evaluated until 48 h after exposure. Efficacy was observed as early as 12 h after exposure, with a significant reduction of live mosquito counts in both groups treated with sarolaner until day 14. At the 24-h timepoint, a significant reduction was observed for all days in the Simparica-treated group and until day 21 in the Simparica Trio-treated group. By 48 h, the mosquito counts were significantly reduced for all days in both treatment groups. The results of study 2 confirmed the high efficacy, with significant reduction for 35 days for the Simparica-treated group and for 28 days for the Simparica Trio-treated group at the 24-h timepoint. As in study 1, live mosquito counts at the 48-h timepoint were significantly reduced for 35 days in both treated groups, with > 90% efficacy when compared to the untreated control group for 35 days in the Simparica-treated group and for 21 days in the Simparica Trio-treated group. Mosquitoes allowed to feed for 1 h on Simparica or Simparica Trio-treated dogs are thus exposed to lethal doses of the active insecticidal compound, sarolaner, in these products. A more rapid speed of kill was observed shortly after treatment compared to later in the monthly treatment period, which aligns with the pharmacokinetics of sarolaner which show a high and rapid increase shortly after treatment and a decline towards the end of the treatment period [3]. The data thus confirm that, after a single administration on day 0, the quantity of sarolaner present in the dog’s blood was sufficiently high to kill between 90 and 100% of the fed female mosquitoes throughout an entire month. In a previous study, the efficacy of afoxolaner was evaluated at 24 h post-exposure, with high efficacy up to day 7 and less than 90% efficacy from day 15 onwards [5].

Given the lack of an anti-feeding effect, it is not expected that treatment with a systemic product such as sarolaner directly impacts the transmission of mosquito-borne diseases. Nevertheless, the high efficacy of sarolaner within 48 h after a blood meal is relevant, as *A. aegypti* female mosquitoes start laying eggs 48 h after a blood meal [8]. The impact on egg production was examined in study 2, which indeed demonstrated that Simparica enables the complete suppression of egg production for 35 days, whereas Simparica Trio was able to prevent and/or greatly reduce egg production for 35 days after a single treatment. In view of the repeated feeding behaviour of *A. aegypti* on the same host (multi-blood feeding), or on different hosts (multi-host feeding), often as frequently as every 2–3 days, the high efficacy within 48 h after one exposure to sarolaner implies that both multi-blood feeding and multi-host feeding are prevented. The high insecticidal efficacy of sarolaner has the potential to reduce the mosquito population in the microenvironment around the treated animal by killing the mosquitoes prior to a new feeding bout and by preventing, or suppressing, the females’ egg production. As such, sarolaner can have an indirect effect on the rate of vector-borne pathogen transmission, including that of canine heartworm, within that microenvironment, as well as a direct effect with respect to the negative effects of mosquito bites, such as irritation and allergic reactions. These potential benefits need to be further examined through modelling or a simulated household environment study.

## Conclusions

A single oral dose of Simparica or Simparica Trio provides high efficacy against mosquitoes in dogs within 24–72 h after exposure for an entire month, as measured by the reduction in live fed-mosquito counts and egg production.

## Data Availability

Data can be requested from the corresponding author.
